# *In vitro* effect of hydroxychloroquine on pluripotent stem cells and their cardiomyocytes derivatives

**DOI:** 10.3389/fphar.2023.1128382

**Published:** 2023-07-12

**Authors:** Michelle Vanessa Kamga Kapchoup, Jürgen Hescheler, Filomain Nguemo

**Affiliations:** Centre for Physiology, Faculty of Medicine and University Hospital Cologne, University of Cologne, Cologne, Germany

**Keywords:** hydroxychloroquine, stem cells, proliferation, differentiation, cardiotoxicity

## Abstract

**Introduction:** Hydroxychloroquine (HDQ) is an antimalarial drug that has also shown its effectiveness in autoimmune diseases. Despite having side effects such as retinopathy, neuromyopathy and controversial cardiac toxicity, HDQ has been presented and now intensively studied for the treatment and prevention of coronavirus disease 2019 (COVID-19). Recent works revealed both beneficial and toxic effects during HDQ treatment. The cardiotoxic profile of HDQ remains unclear and identifying risk factors is challenging.

**Methods:** Here, we used well-established cell-cultured to study the cytotoxic effect of HDQ, mouse induced pluripotent stem cells (miPSC) and their cardiomyocytes (CMs) derivatives were exposed to different concentrations of HDQ. Cell colony morphology was assessed by microscopy whereas cell viability was measured by flow cytometry and impedance-based methods. The effect of HDQ on beating activity of mouse and human induced pluripotent stem cell-derived CMs (miPSC-CMs and hiPSC-CMs, respectively) and mouse embryonic stem cell-derived CMs (mESC-CMs) were captured by the xCELLigence RTCA and microelectrode array (MEA) systems.

**Results and discussion:** Our results revealed that 20 µM of HDQ promotes proliferation of stem cells used suggesting that if appropriately monitored, HDQ may have a cardioprotective effect and may also represent a possible candidate for tissue repair. In addition, the field potential signals revealed that higher doses of this medication caused bradycardia that could be reversed with a higher concentration of ß-adrenergic agonist, Isoproterenol (Iso). On the contrary, HDQ caused an increase in the beating rate of hiPSC-CMs, which was further helped upon application of Isoproterenol (Iso) suggesting that HDQ and Iso may also work synergistically. These results indicate that HDQ is potentially toxic at high concentrations and can modulate the beating activity of cardiomyocytes. Moreover, HDQ could have a synergistic inotropic effect with isoproterenol on cardiac cells.

## 1 Introduction

Hydroxychloroquine (HDQ) which belongs to the 4-aminoquinoline family, has anti-malarial properties and has also been shown to be effective against rheumatoid arthritis (Mackenzie and Scherbel, 1980; Furst, 1996) and lupus erythematosus (Dubois, 1978), which doses should not exceed the range recommended per day to avoid side effects such as retinopathy. Oral HDQ is quickly taken by the gastrointestinal tract and reaches its maximal peak in blood after approximately 4 hours (Al-Rawi et al., 2018). Moreover, studies on mice designed as an equivalent to human clinical trials showed that the pharmacokinetics of HDQ may be dose-dependent whereas the pharmacodynamics are not (Jackson et al., 2016) suggesting that at a low HDQ concentration, cell efficacy may not be compromised. Studies also demonstrated that the half-life of HDQ elimination is more than 1 month (Browning, 2014) which outcome could be detrimental for the lifetime users because of its accumulation in the vital organs. Indeed, it has been demonstrated that after reaching the blood system, HDQ will be quickly redistributed into many tissues such as the spleen, the heart, the lung, and the liver, which later represent the organ with a higher accumulation range (Liu et al., 2021). However, HDQ has shown prophylactic properties during transplantation by suppressing activity against graft-versus-host disease after allogeneic peripheral blood stem cell transplantation (Fong et al., 2007). In addition to this, it has also been proposed as a new therapeutic option in acute myeloid leukaemia (Folkerts et al., 2017). Other studies revealed that HDQ might increase the bone mineral density of patients suffering from primary Sjögren Syndrome (pSS), nevertheless, *in vitro* studies revealed that HDQ inhibits osteoblast differentiation and mineralization, which the author attributes to the variance between the *in vitro* model that lacks systemic factors stimulating the bone formation and the *in vivo* model, which contain it (Both et al., 2018). Additionally, the interaction between HDQ and heart dysfunction has been debated, with one viewpoint claiming that HCQ has a cardioprotective effect and the other arguing that it has a cardiotoxic impact. For example, some studies have observed proarrhythmic electrocardiographic alterations linked with HDQ therapy, while others have reported antiarrhythmic changes (Mubagwa, 2020). Because of its antiviral properties and its low side effects, HDQ has been proposed as a drug candidate for COVID-19. Although a study reported that HDQ weakens the heartbeat of COVID-19 patients (Wong et al., 2021), A review regarding the outcome of the drug on the virus has been reported (Oscanoa et al., 2020) speculating that HDQ can be offered as a therapy for COVID-19, and adverse drug reactions or arrhythmias incidence might be associated with the previous lifestyle or preliminary disease of the patients or the drug dosage as the author has reported. For this last hypothesis, Garcia-Cremades et al. (2020) recommended optimizing the HDQ doses to better control the arrhythmic events occurring during drug administration and thus promote better patient survival.

If some studies have highlighted the advantages and disadvantages of HDQ and brought more light on the effect of HDQ during treatment, not much is known regarding its *in vitro* effect on cellular level, and less is truly known about its outcome on cardiac activity. Moreover, the hypothesis of correcting the heart beating rate via ß-receptor stimulation after arrhythmia caused by HDQ treatment (Sutanto and Heijman, 2020) requires more investigation.

## 2 Materials and methods

### 2.1 Mouse embryonic and induced pluripotent stem cells cultures

Mouse embryonic stem cells (mESCs) and induced pluripotent stem cells (miPSCs) were used to study the cytotoxicity of HDQ. The mESCs lines were obtained from Wernig and co-workers (Wernig et al., 2007) and miPSCs were generated at our institute as previously described (Fatima et al., 2016). MiPSCs and mESCs were maintained on irradiated mouse embryonic fibroblasts (MEF) in Dulbecco’s Modified Eagle’s Medium (DMEM) (Gibco) supplemented with 15% fetal bovine serum (FBS), 1 × non-essential amino acids, 2 mM L-glutamine, 50 μM 2-mercaptoethanol, and 1000 U/mL Leukemia Inhibitory Factor (LIF; ESGRO, Chemicon International). Murine embryonic fibroblasts were prepared from transgenic C57BL6 mice carrying Neomycin resistance gene mice at embryonic day 14.5 and inactivated by mitomycin C treatment. The cells were passaged regularly at day 2 or 3 by trypsinization (0.05% trypsin/EDTA) and 0.5 × 10^5^ cells were added to a 6 cm dish with pre-plated murine embryonic fibroblasts (0.8 × 105/dish) (Pfannkuche et al., 2009). During cell passaging, the enzymatic reaction was stopped using the same volume of medium and the cells were collected in a 15 mL tube and centrifuged for 2 min at 1,000 rpm at room temperature (RT). Thereafter, cells were re-suspended in 15% culture media (DMEM (1x) + Glutamax, FBS, MEM-NEA(1X), Penicillin-streptomycin (1X), ß-Mercaptoethanol [50 µM), LIF (1U/µL)], counted and plated back onto a 3 cm coated plated. LIF was added to the cell culture to prevent cell differentiation and the culture was kept in the incubator at 37°C and 5% C02 until they are ready to passage.

### 2.2 FACS analysis

The toxic effect of HDQ on miPSCs was investigated through FACS analysis with the red fluorescent nuclear counterstain propidium iodide (PI). To perform this analysis, 0.7 × 10^6^ cells were seeded in a 12-well plate and incubated at 37°C with 5% CO2 for 24 h. The cells were treated the next day with Hydroxychloroquine (HDQ) (at concentrations ranging from 50 µM to 250 µM and incubated for 48 h. After this incubation time, the cells were trypsinized as mentioned above, centrifuged and re-suspended in 250 µL of 0.5% BSA. The cells were collected in FACS tubes and the samples were run with FACS DIVA. Analysis was performed with FlowJo7.

### 2.3 Pluripotent stem cells (mPSCs) differentiation into cardiomyocytes

Mouse iPS cells (miPSCs) and ES cells (mESCs) were passaged as mentioned above, counted and 1 × 10^6^ cells were re-plated on 10 cm Petri dishes in an end volume of 12 mL differentiation media [for 500 mL: 395 mL IMDM, 100 mL FBS, 5 mL MEM-NEA (100X), 5 mL Penicillin-streptomycin, 1 mL ß-Mercaptoethanol (50 mM)]. 200 µg/mL of Ascorbic acid (AA) were added to the media to promote differentiation. The plate was placed on a shaker n an incubator at 37°C and 5% CO2 and left for 2 days to allow the formation of embryoid bodies (EBs). After 48 h incubation time, 800 EBs were splited into new Petri dishes, and cells were allowed to differentiate further for another 5 days without media change. At D7-D9, cells were pooled in a 2:1 ratio, and puromycin was added when approximately 70% of the beating activity was observed (14 mL of differentiation media +50 µg/mL AA). At D12 or D14, EBs were dissociated into single cells for further experiments. These cardiomyocytes were previously characterized by Fatima et al., (2016).

The human induced pluripotent stem cells (hiPSC) and hiPSC-derived CMs (hiPSC-CMS) as previously disclosed were used (Hamad et al., 2019). Briefly, Cardiomyocytes differentiation from hiPSCs (hiPSC-CMs) was achieved in 2D format using modified small molecules and Wnt signalling inhibitors.

### 2.4 xCELLigence real-time cell analyzer experiment

The xCELLigence real-time cell analyzer (RTCA) which uses a non-invasive high-resolution impedance readout was used to assess in real-time the functional activity (attachment, morphology, and rate proliferation and beating rate) of miPSCs growing inside the wells of the E-Plate Cardio 96 (ACEA Biosciences) before and after application of varying concentrations of HDQ.

For proliferation experiment, 25,000 cells were re-plated in each well of on an E-plate coated with 0.1% gelatin and left for at least 2 h in the incubator at 37°C and 5% CO2. Following this first cell attachment step, the E-Plate Cardio 96 was put in the xCELLigence RTCA Cardio reader (ACEA BIOSciences Inc., San Diego, United States) and the effect of HDQ on proliferating iPSCs was tracked for 10 days. Cell proliferation is determined by the cell index (CI) value, which increases or decreases with the level of cell adhesion corresponding to the cell growth. Therefore, the CI could indicates cell viability or cell death in wells before and after drug treatment.

To measure the effect of HDQ on cardiac beating activity, 32,000 to 35,000 mES cell-derived cardiomyocytes (mESC-CMs) were plated on each well of E-Plate Cardio 96 (ACEA Biosciences) pre-coated with 1% gelatin, and placed in an incubator at 37°C and 5% CO2 for at least 3 days to allow cells attachment and syncytium formation. After the generated monolayer of CMs was treated with different concentrations of HDQ. Treatments for long-term exposures were repeated every 2 days until analysis. The raw data of CI measurements were taken every 10–15 min and beating activity as well as the amplitude were acquired and analyzed using the RTCA Cardio Software 1.0, sigmaPlot 8.0, and CorelDraw 2020.

### 2.5 Field potential (FP) recordings

For extracellular field potential (FP) recordings, a microelectrode array (MEA) setup with a Multichannel Systems 1060-Inv-BC amplifier and data collecting system (Multichannel Systems, Reutlingen, Germany) was employed. The MEA electrode is made up of 60 titanium-nitride electrodes with gold contacts (0, 3 µm in diameter) placed in an 8 × 8 electrode grid with a 200 m inter-electrode spacing. FPs can be captured at up to 50 kHz sampling rate. In serum-free IMDM medium, standard measurements were performed at a sampling rate of 2 kHz. On plasma-cleaned planar MEAs, freshly cut ventricular slices were plated. The Multielectrode chip was pre-coated with 40 µg/mL Fibronectin. miPS cell-derived cardiomyocytes (miPSC-CMs) were re-plated on the MEA plate at a density of 0.25 × 10^6^ and incubated for at least 2 days to allow cell attachment. For human cardiac cells, 40-day-old cardiac derived cells from a healthy donor were re-plated as clusters on Matrigel pre-coated MEA plate and incubated at 37°C and 5% CO2 for at least 5–7 days to allow attachment before FP recordings. FP recording was performed on a warm plate heated at 37°C. Baseline recordings were done for at least 2 min followed by application of different concentrations (applied gradually) of HDQ. Recordings were done for at least 3 min per concentration.

### 2.6 Statistical analysis

Data analysis was performed using Graphpad Prism, SigmaPlot 8.0, FlowJo7, RTCA Cardio 1.0 software, and a cardio QT software that was developed in our laboratory with LabView. The statistical analysis was performed using a *t*-test and repeated measure ANOVA. Dunnett’s *post hoc* test was performed after ANOVA.

## 3 Results

### 3.1 Hydroxychloroquine (HDQ) supports the proliferation of miPSCs at lower concentration

**FIGURE 1:**
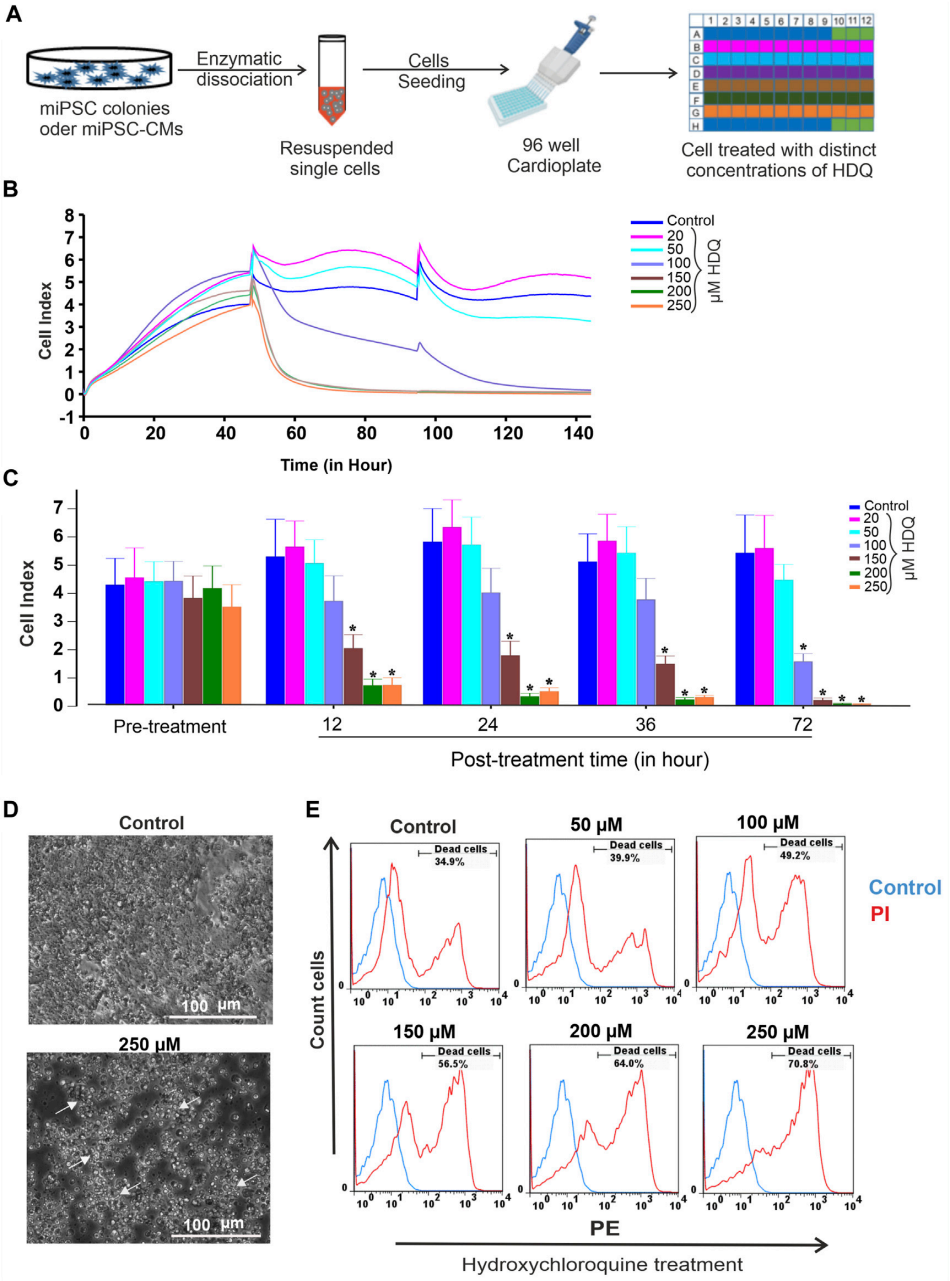
Effect of HDQ on mouse induced pluripotent stem cells (miPSCs). **(A)** Experimental workflow for culturing, dissociating and seeding cells on E-Plate cardio 96 of xCELLigence RTCA System. **(B)** Exemplary recordings showing changes in cell index (CI) in the presence of different concentrations of HDQ as compared to control condition. Cells (25,000 cells/well) were seeded and incubated at 37°C with 5% CO_2_ 2 h before measurement. Different concentrations of HDQ were generally added 24 h post plating. **(C)** Peak cell index values before (pre-treatment) and after treatment with different concentrations of HDQ for 12, 24 36 and 72 h. **(D)** Representative bright field microscopic images of control and 250 µM HDQ-treated cells after 48 h exposure. **(E)** Representative flow cytometry histogram showing the effect of different concentrations of HDQ on miPSCs after 48 h exposure. Data are presented as the mean ± SEM of three independent experiments. *p < 0.05 significantly different compared with control.

To access the toxicity effect of HDQ on undifferentiated miPSCs, cell culture was done on embryonic feeder cells with LIF supplement as described previously (Pfannkuche et al., 2009). The undifferentiated cells were dissociated and re-plated at a density of 25,000 cells/well on an impedance plate coated with 0.1% gelatin and monitored through an impedance system as schematized below (Figure 1A) and cytotoxicity has been further validated via the FACS system. In our experiments, the 20 µM and 50 µM concentration ranges show a positive effect on cell proliferation (Figure 1B), with more pronounced over time for the 20 µM concentration. Treatment of cells with 100 µM showed less toxicity in the first 36 h, and concentrations above this range had a significant concentration-dependent toxic effect, characterized by pronounced cell apoptosis (Figures 1B, C). Therefore, appropriate control of the HDQ concentration during the treatment of degenerative diseases could be beneficial for patients. Additionally, the cytotoxic effect of HDQ was further examined by examining cells monolayer morphology. Data revealed that the cells treated with 250 µM showed significant decrease number and presenting remarkable reduction of monolayer area (Figure 1D). HDQ toxicity was further validated through FACS analysis (Figure 1E). In pharmacology, half-maximal inhibitory concentration (IC50) is widely used to measure a drug’s efficacy and provides information about the concentration at which half of the maximum inhibitory efficacy of a drug is achieved. After calculating the maximal control and normalizing the data, we obtained an IC50 of 100 µM. However, it is also to consider that this concentration may vary between distinct cell types.

### 3.2 Low HDQ concentrations increase proliferation while high HDQ concentrations inhibit inotropic activity in murine cardiac cells

**FIGURE 2:**
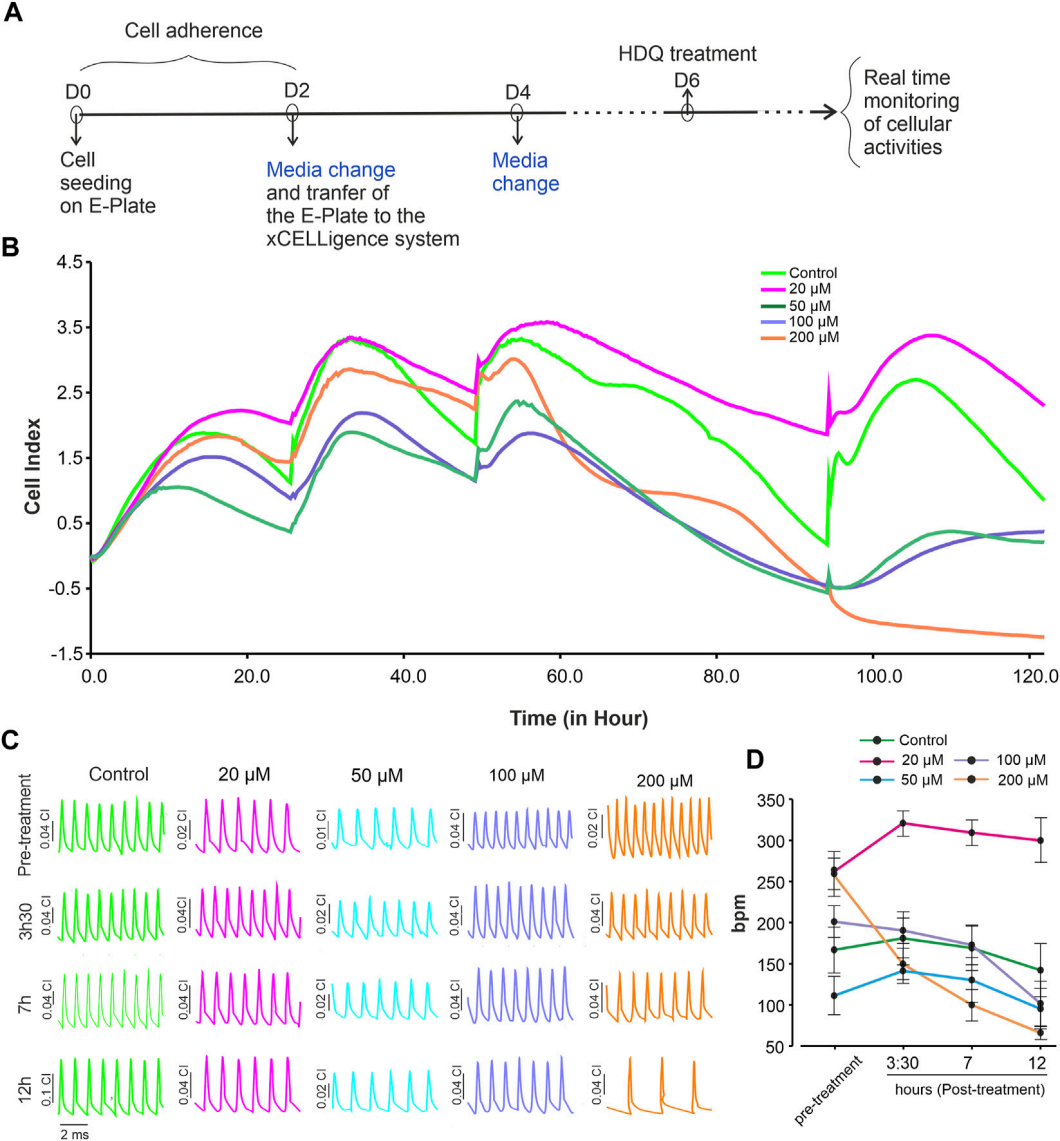
Dynamic monitoring the effect of HDQ on mESC-CMs using RTCA cardio system. **(A)** Experimental workflow used to examine the effect of HDQ on mouse ES cell-derived cardiomyocytes (mESC-CMs) proliferation and beating activity. **(B)** Representative kinetics showing change in cell index in the absence (control) or presence of increasing concentration of HDQ. **(C)** Representative images of changes in beating activity of cardiomyocytes at different time points treated or not (control) with different concentrations of HDQ. **(D)** Average of beating rate (1/minute) of mESC-CMs recorded at indicated time points before- (pre-treatment) and after treatment with different HDQ concentrations at different time points. Data are shown as mean ± SEM of 6 wells each from three independent experiments. *p < 0.05 vs control.

Arrhythmias have been reported after HDQ treatment (Wong et al., 2021), whereas other studies reject this hypothesis mentioning inappropriate use of the drug (Garcia-Cremades et al., 2020). To gain more insight into this issue, real-time recording of beating activity and proliferation of murine cardiac cells in the presence of HDQ at different concentrations was performed for 48 h (Figure 2A). The result shows that 20 µM HDQ increases the proliferation cells whereas the concentration ≥50 µM, induce apoptosis (Figure 2B). Additionally, we observed that at the concentrations of 20 µM and 50 µM, the beating activity did not significantly changed during the first 12 h (Figures 2C, D). Since 50 µM HDG impeded the proliferation as revealed by let CI, we further analysed the effect of HDQ at concentration ≥50 µM on the beating activity of CMs. At a concentration above 50 µM, the beat rate of CMs decreased with time, with pronounced effect under 200 µM HDQ. However, 20 µM HDQ shown slight increase of beating activity of cardiac cells after exposure. The effect was more pronounce after 3 h and seem to disappear after 12 h, suggesting at least in part an activation and deactivation of some signalling pathways regulating the beating activity of the cells.

### 3.3 Effect of HDQ and isoproterenol on the field potential recording of human and mice cardiac cells

**FIGURE 3:**
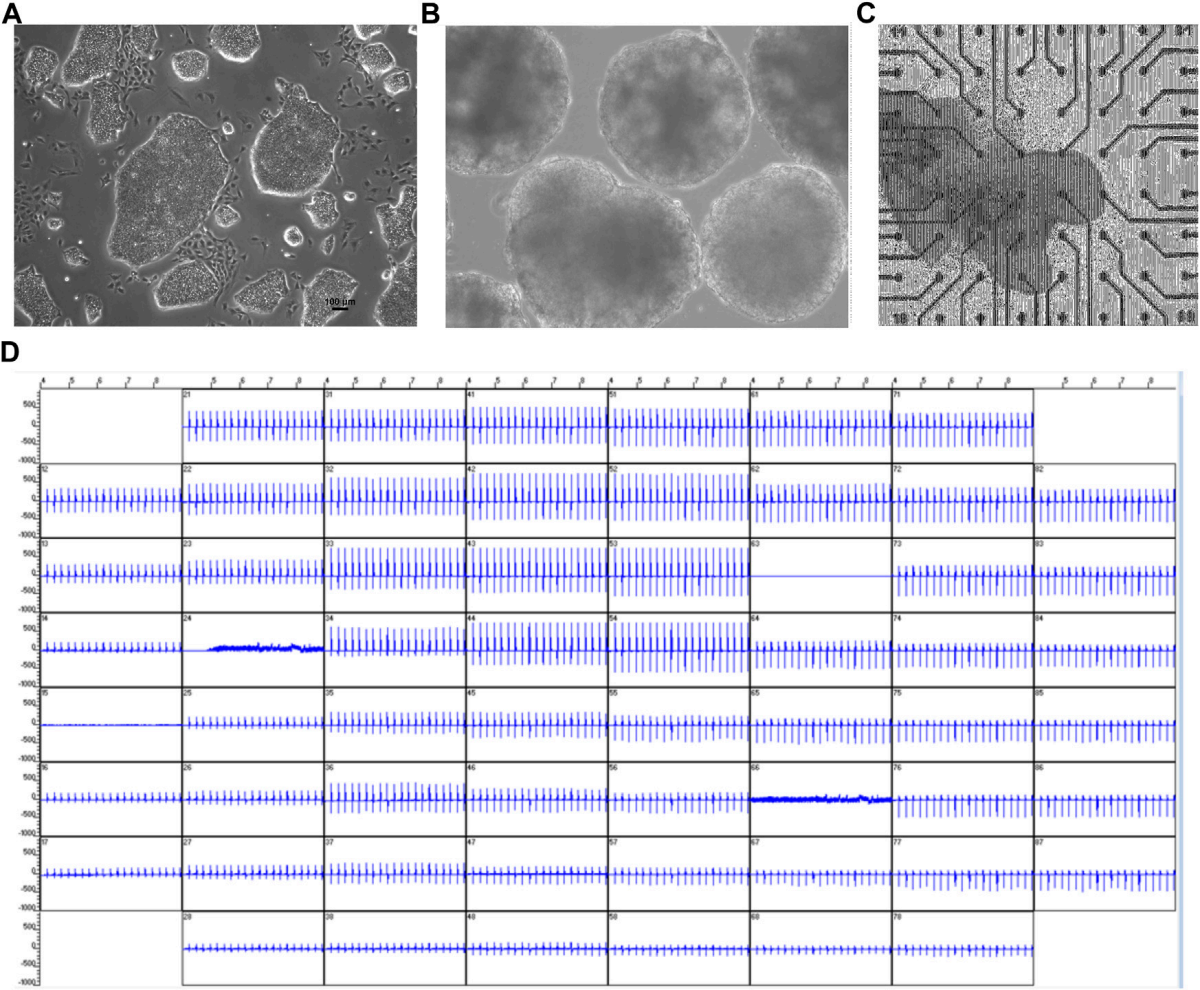
Multielectrode arrays recordings of spontaneously beating EBs/cardiac cluster and effect of HDQ. **(A,B)** Microscopy images of mES cells (mESC) colony **(A)** and EBs **(B)** at day 14 of differentiation. **(C)** Viable and spontaneously beating mESC-derived cardiac cluster attached to MEA chamber. **(D)** Representative extracellular field potential (FP) of beating cluster of miPSC-CMs recordings from all 60 electrodes of MEA plate.

**FIGURE 4:**
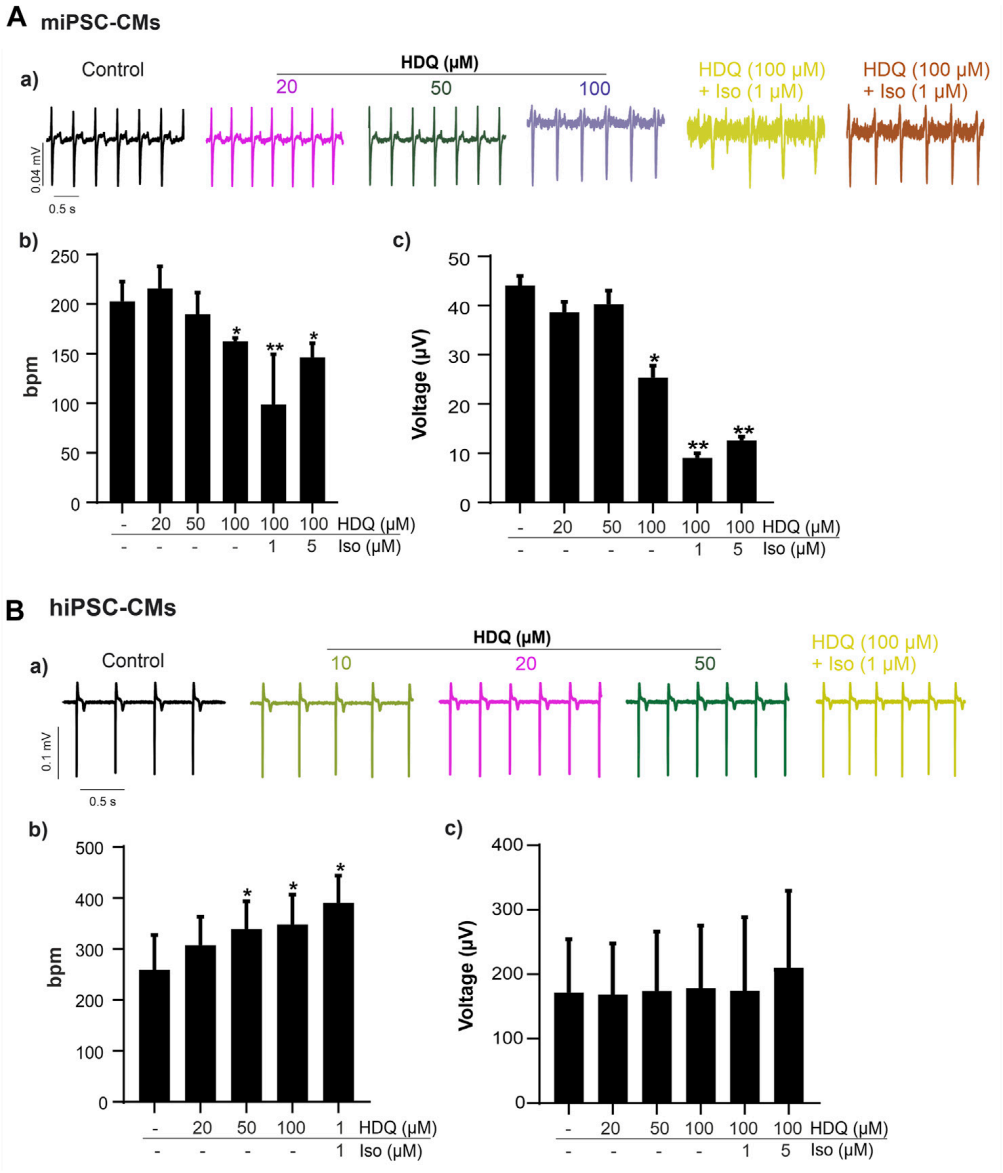
Effect of HDQ and ß-adrenergic receptor agonist on field potential recorded on beating cluster of miPSC-CMs **(A)** and hiPSC-CMs **(B)** using MEA system. Representative field potential **(a)** traces demonstrating effects of ß-adrenergic agonist isoprenaline (ISO) on clusters of miPSC-CMs **(A)** and hiPSC-CMs **(B)** pre-treated with HDQ as indicated. Concentration of both compound, ISO and HDQ were added gradually. The exposure time was at least 3 min for each concentration. Statistical analysis of FP frequencies **(b)** and voltage **(c)** in MEA recordings of miPSC-CMs **(A)** and hiPSC-CMs **(B)**. Data are shown as mean ± SEM of three independent experiments. * *p* < 0.05, ** *p* < 0.005 vs. control (−/−: absence of drug).

**FIGURE 5:**
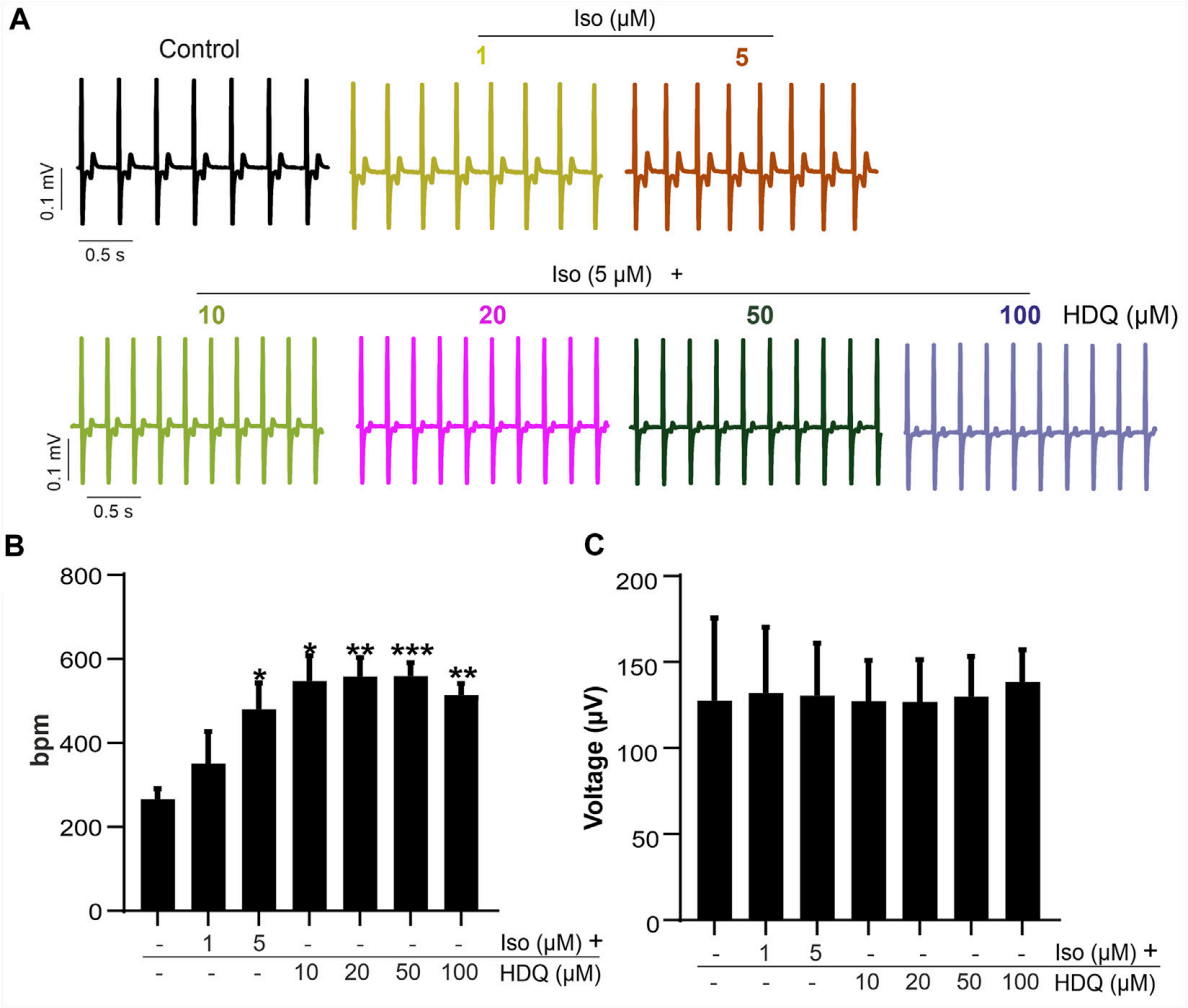
Effect of HDQ on field potential of hiPSC-CMs pre-stimulated with increase concentration of ß-adrenergic receptor agonist. **(A)** Representative field potential traces and frequencies from MEA analysis of pre-stimulated beating hiPS cell-derived CMs (hiPSC-CMs) cluster exposure to different concentrations of HDQ added gradually. Concentration of ISO and HDQ were added gradually for at least 3 min exposure time for each concentration. **(B,C)** Statistical analysis of FP frequencies **(B)** and voltage **(C)** recorded after treatment as indicated. Data are shown as mean ± SEM of three independent experiments. * *p* < 0.05, ** *p* < 0.005*,* *** *p* < 0.0005 vs. control (−/−: absence of drug).

Since ß-adrenergic agonist has been used to correct arrhythmia induced by HDQ (Sutanto and Heijman, 2020). In this study, we performed a Multielectrode array experiment to better understand their pharmacokinetic and pharmacodynamics. For these experiments, beating cluster of miPSC-CMs or hiPSC-CMs were plated on 1% gelatin of Fibronectin microelectrode arrays (MEAs) dish (Figures 3A–C). The analysis of the field potentials (FPs) signal were performed (Figure 3D). Using miPSC-CMs, we observed that a concentration of 20 µM of HDQ tended to enhance the beating activity (Figure 4A-a). However, high concentrations (100 mM) decreased the cell beating rate (Figure 4A-b) and the voltage (Figure 4A-c) of hiPSC-CMs in a concentration-dependent manner as compared to controls. At high concentration of Iso (5 µM), we were able to observe a trend towards restoration of the beating rate of these cells, while the 1 µM concentration did not. Using human-induced cardiomyocytes (hiPSC-CMs) (Figure 4B), we observed increase in beating activity under HDQ (Figures 4Ba, b) in concentration dependent manner, which effect was further enhanced by the application of isoproterenol (Iso). No change in the voltage (Figure 4B-c) of hiPSC-CMs was observed. This observation was also true when hiPSC-CMs clusters were pre-treated with Iso (Figures 5A, B). In both cases, the result was statistically significant suggesting that HDQ and Iso may have a synergistic ionotropic and chronotropic effects on hiPSC-CMs at tested concentrations. However, the voltage of hiPSC-CMs remains unchanged (Figure 5C). These observations challenged the hypothesis of drug synergy between HDQ and Iso observed with hiPSC-CMs and underline the importance of taking into consideration the type of cells used during *in vitro* drug essays.

## 4 Discussion

Hydroxychloroquine is widely used because of its less toxic effect for various therapeutic approaches such as Blood (Folkerts et al., 2017) or lung (Li et al., 2018) cancer, and autoimmune diseases (Furst, 1996), and it has even been proposed as a prophylactic drug during allogeneic transplantation (Fong et al., 2007). Recently it has been used to treat COVID-19 patients. Although showing minimal toxic effect, recent studies revealed that a prolonged utilisation of this drug or improper doses could cause retino- neuro- and/or myopathy (Phillips and Chun, 2014; Yusuf et al., 2017; Stokkermans et al., 2021). Due to its wide use by physicians, we aim in this study to further evaluate the effects of this drug on induced pluripotent stem cells and derived cardiomyocytes (miPSC-CMs) to provide further insight into the therapeutic properties of this drug. In our study, we found that at a concentration of 20 µM, HDQ supports cell proliferation in miPSCs over time, and that concentrations between 50 µM and 100 µM do not have a significant impact on the cell’s proliferation for the first 36 h. However, Concentrations above 100 µM are highly toxic to cells in a time-dependent manner. The fact that HDQ can promote cell proliferation could explain the high bone mineral density (BMD) of some patients under HDQ treatment (Lakshminarayanan et al., 2001; Mok et al., 2005; Both et al., 2016), suggesting that this drug may have a protective effect or be beneficial for patients with low BMD. Nonetheless, although cell proliferation has beneficial effects for the host, it can also be lethal, as uncontrolled cell growth can lead to other complications such as cancer (Yang et al., 2014). Liu et al., (2020) calculated the IC50 value of HDQ in their investigation with African green monkey kidney VeroE6 cells to be 249.50 µM, which differs from our estimate of 100 M. However, the technique as well as the cell type utilized here to test the cytotoxicity of the medication are dissimilar, which might explain the rationale for this shift. The work by Yang et al., (2020) validates the latter assertion by establishing that cell types have a varied susceptibility to the chemical 4-aminoquinoline. We discovered, like this author, that a concentration greater than 100 µM had a high cytotoxic impact after a 24-h incubation.

HDQ therapy has been associated with cardiotoxicity that might be fatal for some patients after long-term therapy (Joyce et al., 2013). However, using mouse cardiomyocytes, we were able to observe that 20 µM HDQ enhances cell proliferation and does not appear to stimulate cardiac contractility. Although we could observe a transient increase in cardiac activity after about 3 h of treatment, we could not associate it directly with HDQ since the control result also shows an increase in beating rate of the cluster of miPSC-CMs at the same time point. The positive inotropy transient effect observed here could be attributed to mechanical stimulation due to the change of medium, which could affect the cell activity for some time. Furthermore, it has been shown that HDQ can play a protective role during ischemia by increasing ERK1/2 phosphorylation (Bourke et al., 2014) suggesting that this drug might be beneficial for patients at a certain concentration. But, the combination of this drug with other medicine such as Azithromycin, which is an antibiotic, may increase the risk of cardiac arrest (Wong et al., 2021). In the fight against SARS COVID-19, it has been shown that HDQ at low concentrations can effectively inhibit the virus (Liu et al., 2020). In fact, in an *in vitro* assay of HDQ and SARS-CoV-2, *Liu et al.* observed that the maximum 50% effective concentration to inhibit the virus is between 4.51 µM and 12.96 µM. This implies that when treating the disease based on HDQ therapy, it is critical to tailor the doses of the drug on the one hand and to avoid using it on patients already showing signs of heart failure on the other hand. Additionally, parameters such as the patient’s history and the severity of the disease must be taken into account. Indeed, a study of patients suffering from systemic lupus erythematosus (SLE) revealed that during medical assistance, physicians should adapt the treatment to severity of the disease for better treatment efficacy (Costedoat-Chalumeau et al., 2006). However, it is also important to note that long-term treatment with HDQ can lead to eye damage and that toxicity is independent of age, weight, or daily dose (Wolfe and Marmor, 2010), making it important to continuously monitor the treatment of lifelong users.

At a concentration of 50 µM, the viability of CMs is affected over time, suggesting that although this concentration is less toxic to stem cells as discussed above, its toxicity to cardiac cells is evident. At the IC50, which corresponds to 100 µM is this study and at concentrations above, HDQ significantly induces apoptosis of cardiac muscle cells and causes negative inotropic activity. Therefore, we suggest that the drug safety should be evaluated on both stem cells and specialized cells such as CMs in order to have a better overview of its results and to better estimate the safe concentration range for patients. ß-adrenergic agonists have been used to restore the cardiac rhythmicity to normal after arrhythmia following the intake of HDQ (Chen et al., 2006; Merino et al., 2020). Using murine derived-cardiac cells, we were able to test this hypothesis. Indeed, we found that 5 µM of Iso could reverse the effect of HDQ and increase the beating rate. However, 1 µM did not influence the action of the HDQ on CMs derived stem cells. Therefore, we hypothesized that a high concentration of Iso is required to restore arrhythmia events caused by HDQ. Field potential recordings of murine cardiac cells further confirm the negative inotropic effect of HDQ from a concentration of 100 µM as mentioned earlier in our impedance experiment. This toxicity is also verified by a reduction in the amplitude of contractility of these cells. Using Human-induced cardiomyocytes, HDQ showed the opposite effect compared to mouse cells. Indeed, in hiPSC-CMs, HDQ increased the beating frequency in a concentration-dependent manner, and this effect was enhanced after addition of isoproterenol. Therefore, we investigated whether both drugs could have a synergistic effect on these cells, and performed the same experiments, but this time we started treating the hiPSC-CMs with Iso followed by HDQ during the MEA measurement. We observed that whether we start with Iso or HDQ, each drug supports the positive inotropic effect of the other in a concentration-dependent manner, suggesting that both drugs might have a synergistic effect which opposite the above-mentioned studies and our experiment on murine cardiac cells. We, therefore, hypothesized that the model used in our study to evaluate the effect of these drugs on hiPSC-CMs may not be accurate or that the human cardiac cells are not sufficiently mature to replicate the results published to date. Nevertheless, it is also known that *in vitro* studies do not offer all the prerequisites that need to be fulfilled to meet the expectation of what happens *in vivo*. Another explanation for these results could be redirected to the complexity of the regulatory mechanism of these drugs and experiment regrouping a better physiological approach could be crucial. Indeed, bradycardia has been mainly described as a side effect of HDQ treatment (Chen et al., 2006; Garcia-Cremades et al., 2020; Tripathy et al., 2020), which was not our observation using human-derived cardiac cells. The difference in this observation could also be due to the concentrations used in our experiment, which are above the therapeutic dose (Garcia-Cremades et al., 2020; Jordaan et al., 2021). Indeed, using adult human primary cardiomyocytes, Jordaan et al., (2021) observed that HDQ concentrations of HDQ well above the therapeutic dose could be responsible for the proarrhythmic effect. However, the side effects of HDQ on cardiac cells and the treatment with isoproterenol after HDQ-induced arrhythmia require further investigation.

In conclusion, we could show that HDQ can cause cell damage at a certain concentration range. However, an appropriate concentration of the drug could promote stem cells and cardiomyocytes proliferation, which could be beneficial for patients. Wherever, further studies on the effect of HDQ on cardiomyogenesis as well as on different cell lines are needed to validate this hypothesis and to determine the appropriate concentration range necessary for normal cell proliferation, which might contribute to the application of tissue repair therapy or assist in the therapy of patients with low BMD. Moreover, we advise careful use of this drug in children to avoid overdose as it may induce cardiac toxicity. In this study, we were also able to show that HDQ and Iso could have a synergistic effect and that the type of cells used *in vitro* or the technique employed to assess the effect of a drug can give distinct results.

## Data Availability

The raw data supporting the conclusion of this article will be made available by the authors, without undue reservation.
